# Overcoming challenges with implementing the Get-To-Know-Me Board to humanize ICU care: a quality improvement project

**DOI:** 10.3389/fmed.2026.1777312

**Published:** 2026-02-26

**Authors:** Joon Yong Moon, Natalie Tanzy, Natasha Schwartz, Amelia Barwise, Lioudmila Karnatovskaia, Ognjen Gajic, Sumera Ahmad

**Affiliations:** Mayo Clinic, Rochester, MN, United States

**Keywords:** critical care, Get-To-Know-Me Board, humanizing, ICU, quality improvement

## Abstract

The intensive care unit (ICU) is a highly disease-focused environment where patients are often perceived primarily through their diagnoses rather than as individuals. The Get-To-Know-Me Board (GTKMB) was introduced as a humanizing tool to highlight patients’ personal attributes and foster person-centered care. Despite being found valuable in supporting humanized caring and fostering communication, completion rates at our institution remained low. Through a stakeholder-driven quality improvement initiative, we tested sequential interventions including reminders and checklists, visual magnets, and educational pamphlets to improve and increase the use of the GTKMB. These measures produced modest gains, with reminders and checklists yielding the most notable improvement. Barriers to using the GKTMB included unclear ownership, timing, and inconsistent integration into workflow. Our experience underscores both the promise and limitations of humanizing interventions in critical care. Sustainable implementation requires multidisciplinary buy-in and integration into daily routines. Whereas humanizing tools such as the GTKMB should be evaluated for their impact on meaningful patient outcomes, such outcomes are not clearly established; therefore, we embark on the necessary first step of a method of implementation.

## Introduction

### Background

The intensive care unit (ICU) is a highly disease-focused environment where patients are often perceived primarily through their diagnoses rather than as individuals (1, 2). This depersonalization can exacerbate patient’s distress, impair communication and contribute to clinician burnout (3, 4). Humanizing strategies, such as open visiting hours, narrative interventions, and ICU diaries, have demonstrated the ability to foster trust and strengthen therapeutic relationships (5). The Patient Dignity Inventory demonstrated how structured approaches can preserve personhood in serious illness (6).

The Get-To-Know-Me Board (GTKMB), introduced in 2006, is a low-cost communication tool designed to capture personal details such as nicknames, hobbies, values, and family information (7–9). Survivors of critical illness and their families have commented that the GTKMB helped to preserve their personhood and foster communication with clinicians during the COVID-19 pandemic (9, 10). Multidisciplinary ICU clinicians also expressed support of the intervention where it enables communication, helps guide care and supports humanized care (8, 11). Similar initiatives, such as the Canadian Footprints Project have reported completion rates near 50% (12).

### Local problem

Despite this value of the GTKMB learnt from previous studies, completion of the GTKMBat our institution’s Medical Intensive Care Unit (MICU) remained far below reported benchmarks. Clinician stakeholders have shared barriers in implementation such as lack of visibility and ownership in completion of the GTKMB, which prompted Building Relationships in Daily Care through GTKMB Engagement (BRIDGE) Qualitative Improvement (QI) initiative.

### Intended improvement

The project aimed to increase GTKMB utilization in the MICU by addressing barriers previously identified by stakeholders. Our rationale was that embedding the GTKMB into daily workflows, reinforcing its visibility, and educating patients, families, and staff would improve uptake of the GTKMB.

### Study question

Can targeted, stakeholder-driven interventions increase GTKMB completion rates and enhance staff engagement with this humanizing tool in the MICU?

## Methods

### Interventions

**Figure 1:**
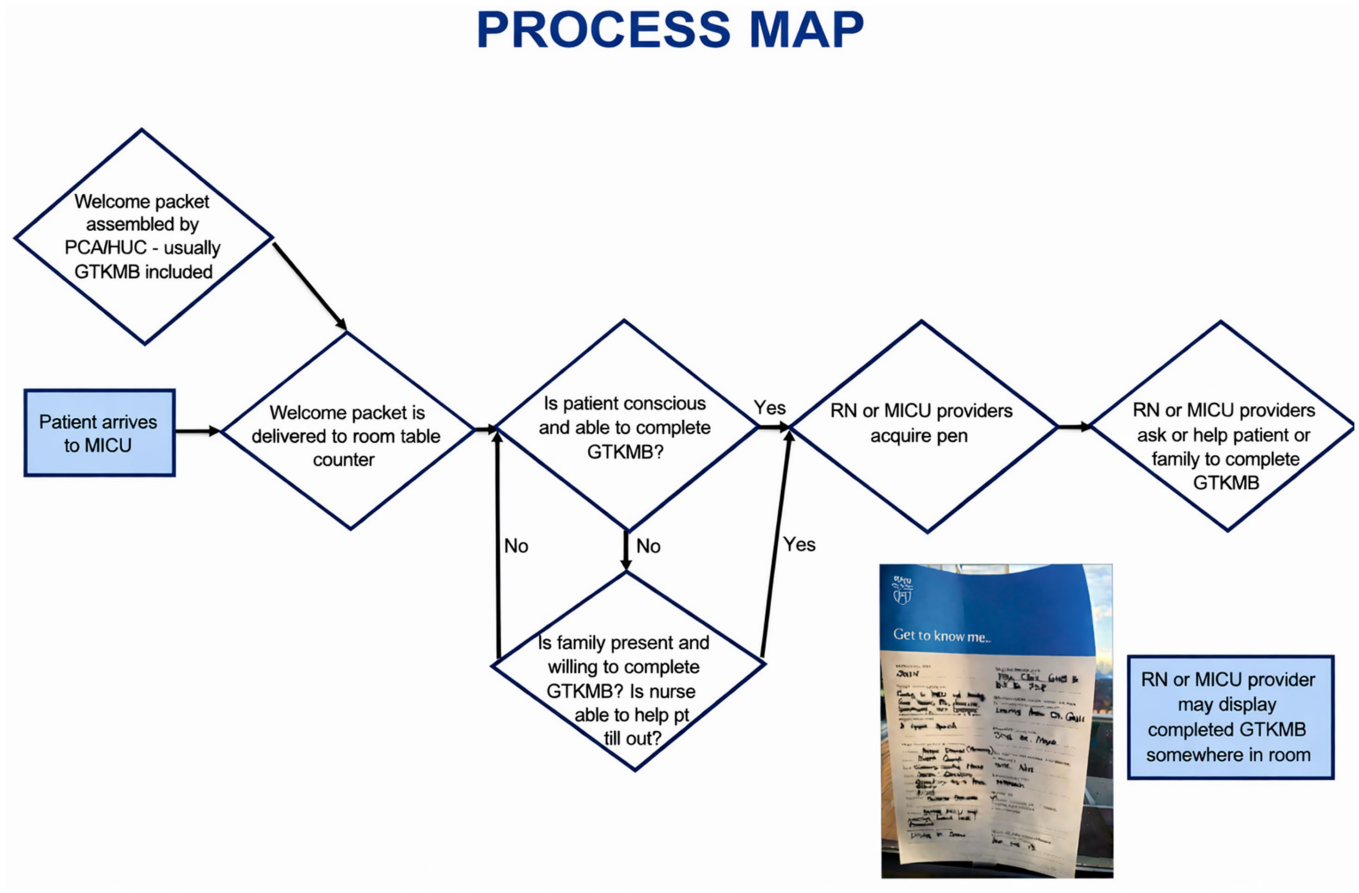
GTKMB process map.

The GTKMB served as the core intervention. Implementation strategies were selected based on stakeholder surveys, process mapping, and feasibility for integration into ICU routines (Figure 1). These strategies were tested through sequential Plan-Do-Study-Act (PDSA) cycles designed to support adoption and integration of the GTKMB into daily practice. Three PDSA cycles were implemented between January and May 2025 (13).

#### PDSA cycle 1: workflow integration

The first intervention focused on embedding GTKMB prompts into existing clinical workflows by incorporating reminders into morning and evening huddles and adding GTKMB prompts to the rounding checklist as part of the A-F bundle (14).

#### PDSA cycle 2: visual cueing

The second intervention introduced a visual cue in the form of a GTKMB magnet placed on patient whiteboards, serving as a visual reminder for staff and families.

#### PDSA cycle 3: patient and family education

The third intervention, developed in collaboration with the Patient Education team consisted of an informational pamphlet explaining the purpose and importance of the GTKMB for patients, families, and staff.

### Study design

This single-center quality improvement project was conducted in the MICU between September 2024 and July 2025, using the DMAIC (Define, Measure, Analyze, Improve, Control) framework (15). During the Define phase, stakeholders (nurses, pharmacists, respiratory therapists, fellows, consultants, and patient care assistants) completed surveys via Google Forms to share their perspectives on implementation barriers. In the Measure phase, baseline GTKMB completion rates were collected on six randomly selected days in September–October 2024. Completion was defined as more than 2 items completed. In the Analyze phase, process mapping and a fishbone diagram were used to identify contributing factors. During the Improve phase, the three sequential PDSA cycles were implemented. Lastly, during the Control phase, reinforcing reminders and educational pamphlets were implemented to help sustain prior efforts.

## Results

### Outcomes and analysis

**Figure 2:**
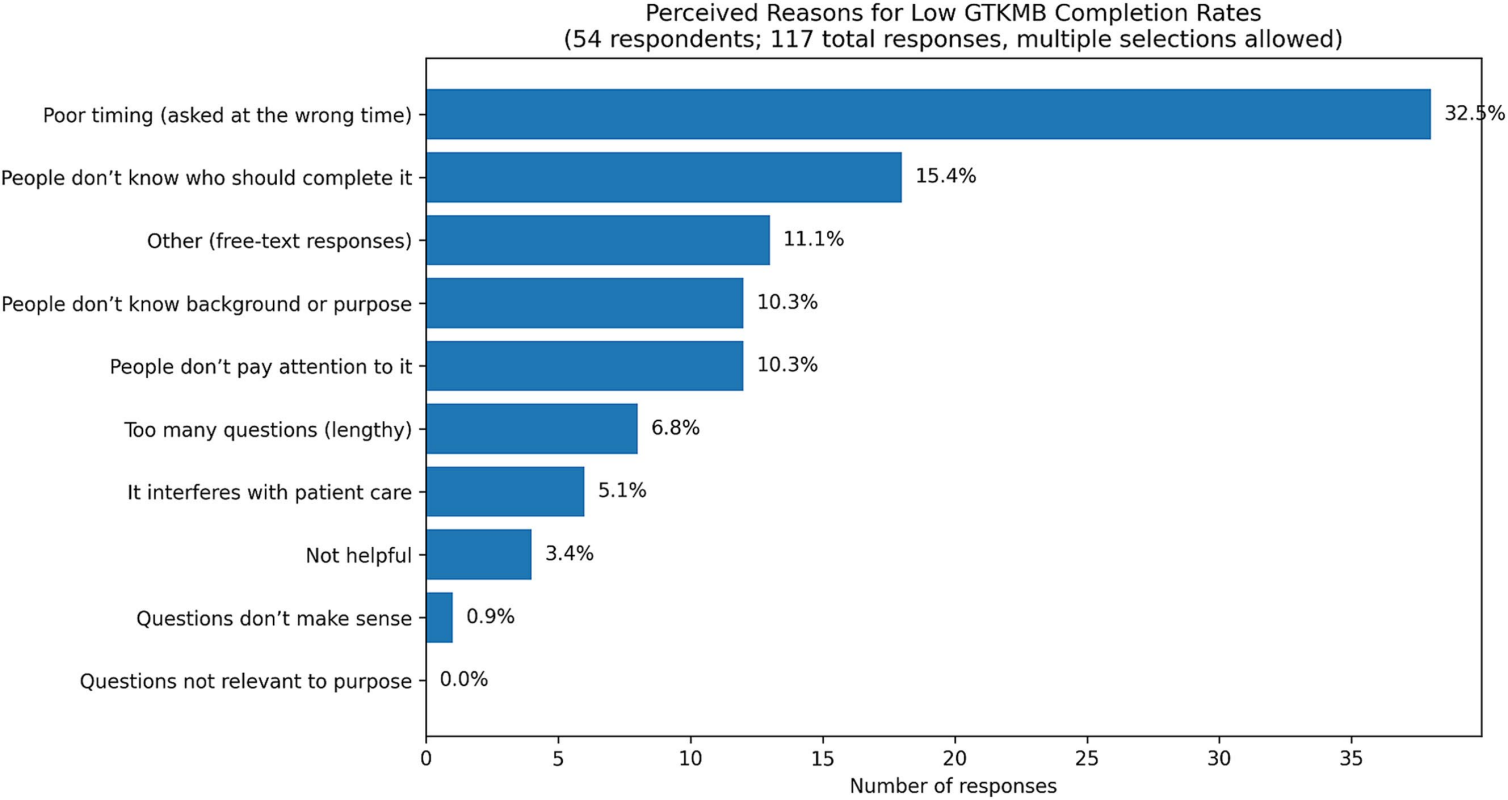
Perceived reasons for low GTKMB completion rates (Within the “other” category, there were 18 individual responses related to poor timing, inability to answer due to confusion or intubation, lack of time, and patient or family unwillingness to respond). Figure revised and refined ChatGPT 5.2 on 22 December, 2025.

Baseline measurements in September and October 2024 showed an average GTKMB completion rate of 23% with daily census ranging between 19 and 27 patients. A comprehensive survey of 54 individual stakeholders with 117 total responses confirmed that poor timing (32.5%), unclear ownership (15.4%), lack of awareness (10.3%), and lack of attention (10.3%) were the biggest barriers to routine use (Figure 2).

**Figure 3:**
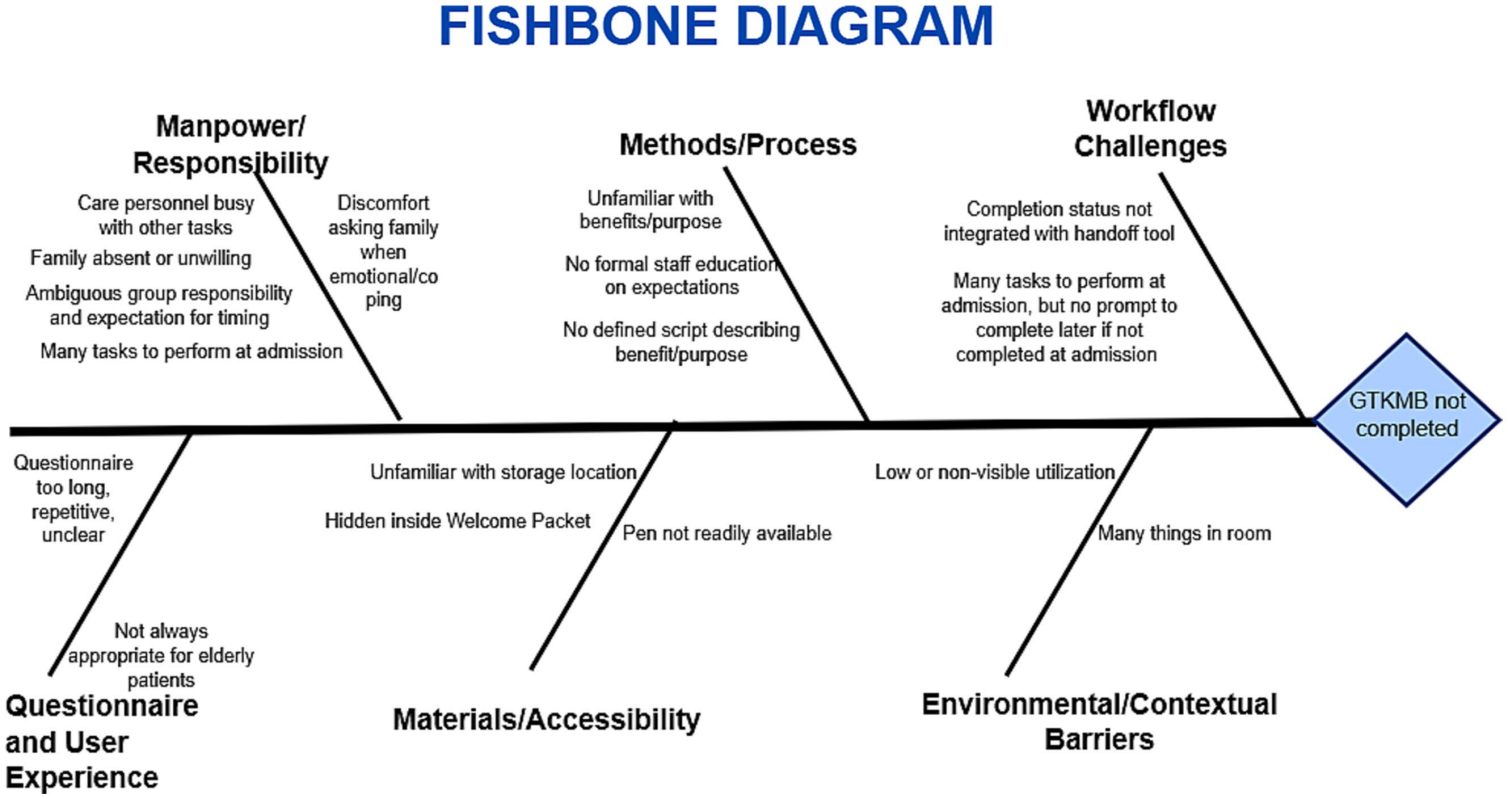
Fishbone diagram. Figure was revised and refined using ChatGPT 5.2 on 22 December, 2025.

Following the initial stakeholder survey, a structured root cause analysis was conducted using a fishbone diagram to identify factors contributing to low GTKMB completion (Figure 3) (16). Contributing causes clustered across multiple domains, included *responsibility* (e.g., unclear ownership), *lack of awareness* (e.g., lack of standardized education, unclear purpose), *workflow challenges* (e.g., poor integration into admission workflows), *materials and accessibility* (e.g., limited visibility of the GTKMB), *user experience* (e.g., perceived redundancy, length, and limited appropriateness for elderly or critically ill patients) and *contextual factors* (e.g., high patient acuity, cognitive overload, and competing bedside demands). This analysis demonstrated that incomplete GTKMB use was primarily driven by structural and workflow-related barriers rather than lack of perceived value, informing the selection of subsequent implementation strategies.

Applying a simplified five-whys logic further clarified how these factors interacted (17). Why was GTKMB completion low? Because it was often not completed during the ICU stay. Why were they not completed? Because initiation was frequently delayed or deferred. Why was the initiation delayed? Because responsibility for completion was unclear. Why was ownership unclear? Because GTKMB use was not embedded into standardized admission or rounding workflows and was inconsistently reinforced by physicians. Why was it not embedded or reinforced? Because relational tools were often viewed as secondary to acute clinical priorities in a high-acuity ICU environment. Together this analysis demonstrated that incomplete GTKMB use was primarily driven by structural and workflow-related barriers rather than lack of perceived value, informing the selection of subsequent implementation strategies.

Following implementation of PDSA Cycle 1 in January 2025, which introduced daily reminders and checklist prompts, the average GTKMB completion rate increased to 47.5%, with a daily census ranging from 22 to 28 patients. In a subsequent survey of 24 stakeholders revealed that 87.5% felt the reminders were helpful in encouraging the GTKMB use.

**Figure 4:**
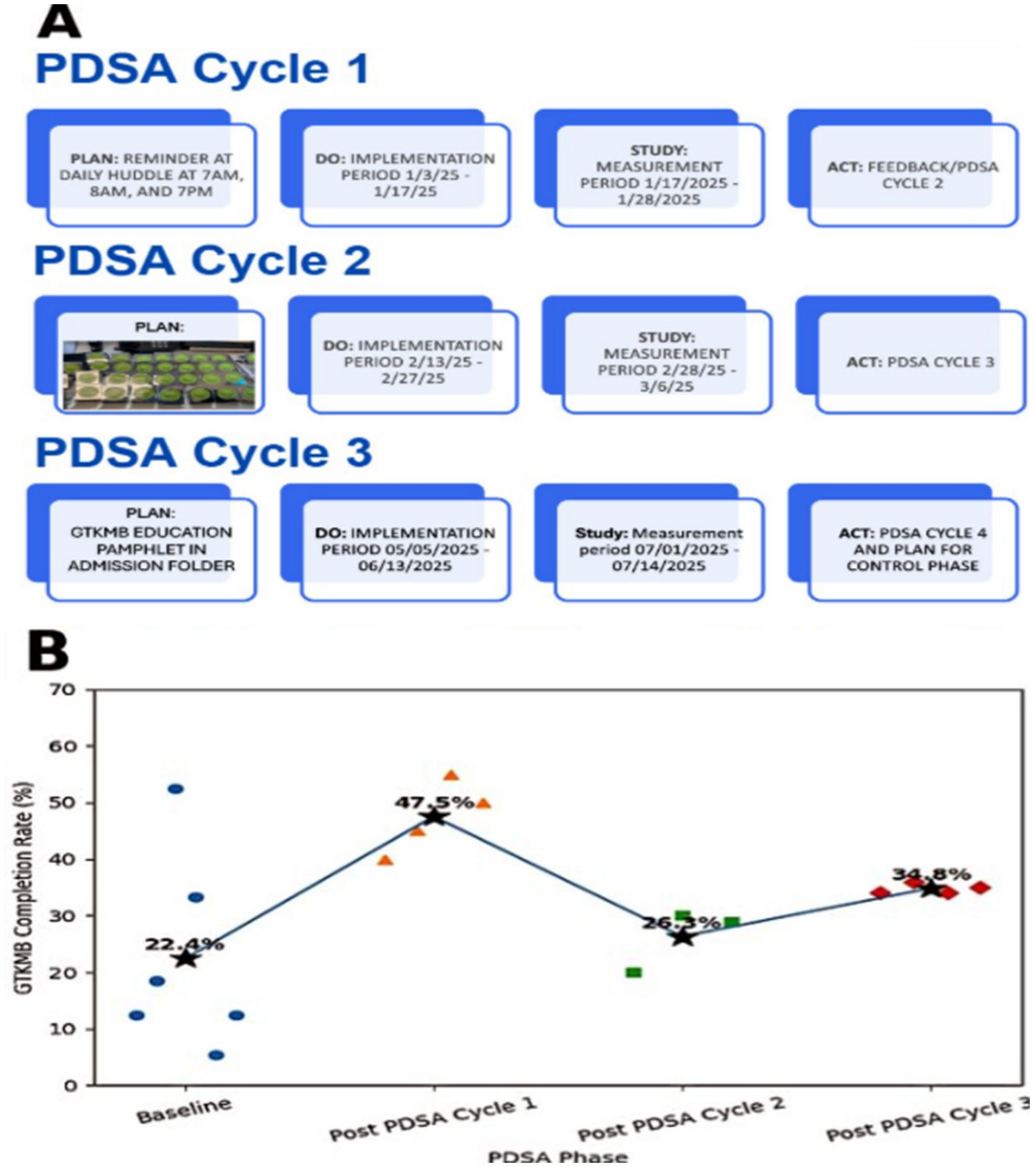
**(A)** Summary of three Plan-Do-Study Act (PDSA) cycles implemented to improve GTKMB completion. **(B)** Associated changes in GTKMB completion rates by study phase. Each data point represents the GTKMB completion on one randomly sampled ay within the phase. Phase-level average completion rates are shown and connected to illustrate changes across PDSA cycles. Blue circles represent baseline measurements. Orange triangles represent post-PDSA Cycle 1 measurements collected between 1/17/2025–1/28/2025. Green squares represent post-PDSA Cycle 2 measurements collected between 2/28/2025–3/6/2025 random days. Red rhombi represent post-PDSA Cycle 3 measurements collected between 7/1/2025–7/14/2025. Star markers indicate the mean completion rate for each phase. Figure revised and refined using ChatGPT 5.2 on 28 January, 2026.

PDSA Cycle 2 was launched in March 2025. During this phase, the average completion rate declined to 26.3%, in part due to wash-out period between PDSA Cycle 1 and 2. The PDSA Cycle 3 was implemented in May 2025, during which an educational pamphlet describing the purpose and importance of the GTKMB was added to the admission folder distributed to all MICU patients. Five weeks after implementation, the average completion rate increased to 34.8%. A summary of the PDSA cycles and the associated changes following each implementation are shown in Figure 4.

Qualitative feedback offered valuable insights. Stakeholders described the Board as an effective tool for engaging with patients on a personal level. However, they also questioned the practicality of it. When it was introduced during acute or emotionally overwhelming phases of care, it felt like a check-box task rather than a genuine effort to connect. Others commented that physicians rarely referred to the GTKMB during rounds. Multiple providers emphasized that while they often form personal connections with patients through direct care, use of the GTKMB required additional effort that felt redundant unless it was championed by the broader multidisciplinary team.

## Discussion

### Lessons and controversies

Our findings align with those reported by the Canadian Footprints Project, which emphasized that humanizing interventions succeed when embedded meaningfully in daily workflows and shared across disciplines (12). Not all feedback was supportive of GTKMB use. Some clinicians felt that personal engagement occurred naturally through direct care and thought that GTKMB created redundancy. Others questioned whether “symbolic communication tools” should be prioritized in a high-intensity ICU when efficiency and workflow pressures are paramount (18, 19). Our experience suggested that the answer depended largely on context. When teams embraced GTKMB as a genuine element of care and shared responsibility for it, the GTKMB fostered meaningful connections. When treated as an isolated task, however, it tended to become a superficial check-box burden.

Some qualitative feedback suggested that the GTKMB felt redundant or artificial to certain clinicians, particularly those who reported forming personal connections through routine bedside interactions. Humanizing care in the ICU care can occur through a range of approaches, including informal clinician-patient conversations, family engagement during rounds, narrative documentation, and structured tools, such as ICU diaries or communication boards (5). Structured tools like the GTKMB may still be valuable in settings where relationship needs to be established between patients, families and clinicians necessary in guiding serious illness conversations and also guiding mulit- disciplinary care such as with nursing and rehabilitation therapists (8, 11). In these contexts, the GTKMB can serve as a shared reference point to support relational continuity.

Although sustainability was partially addressed through sequential PDSA cycles, recurrent barriers, such as unclear ownership and limited clinical engagement, highlight deeper culture and organizational challenges. In a high-acuity ICU, responsibility for relational tools may become unclear when they are viewed as ancillary rather than integral to care. Inconsistent clinician engagement may further signal that GTKMB use is perceived as optional, impeding its integration into routine workflows. These findings suggest that sustainable adoption requires clearly defined ownership, visible leadership endorsement, and integration into team culture.

### Limitation

This study has several limitations. We focused primarily on GTKMB completion as a process measure and did not assess downstream outcome or balancing measures, such as patient or family experience, communication quality, or clinician workload. Given the low baseline completion rate, improving engagement was a necessary first step before meaningful evaluation of these outcomes could be undertaken. Future work should therefore evaluate both patient and clinician-centered outcomes, as well as potential counterbalancing measures, to better define the role of the GTKMB in high-acuity ICU environments.

## Conclusion

The BRIDGE initiative demonstrates that systematic, stakeholder-driven strategies can improve GTKMB uptake, but sustainability requires cultural adoption. Completion rates alone are insufficient to gauge success. Future work should assess outcomes such as patient and family satisfaction, team communication, and clinician well-being. In an ICU dominated by technology and prioritized by disease severity, even a simple GTKMB can redirect attention to personhood, if embraced not as a task, but as care process.

## Data Availability

The raw data supporting the conclusions of this article will be made available by the authors, without undue reservation.
